# Characteristics of quiescent adult neural stem cells induced by the bFGF/BMP4 combination or BMP4 alone *in vitro*

**DOI:** 10.3389/fncel.2024.1391556

**Published:** 2024-05-22

**Authors:** Sutong Xu, Xi Zhang, Zhuoqun Li, Chenming Liu, Qiulu Liu, Huazhen Chai, Hongkai Yao, Yuping Luo, Siguang Li, Chun Li

**Affiliations:** ^1^Stem Cell Translational Research Center, Tongji Hospital, Tongji University School of Medicine, Shanghai, China; ^2^Department of Rehabilitation Medicine, Huashan Hospital, Fudan University, Shanghai, China; ^3^Key Laboratory of Spine and Spinal Cord Injury Repair and Regeneration of Ministry of Education, Orthopedic Department of Tongji Hospital, Tongji University School of Medicine, Shanghai, China; ^4^Tongji University Cancer Center, Shanghai Tenth People’s Hospital of Tongji University, School of Medicine, Tongji University, Shanghai, China

**Keywords:** BMP4, bFGF/BMP4, quiescent neural stem cells, transcriptome, differentiation

## Abstract

Bone morphogenetic protein-4 (BMP4) is involved in regulation of neural stem cells (NSCs) proliferation, differentiation, migration and survival. It was previously thought that the treatment of NSCs with BMP4 alone induces astrocytes, whereas the treatment of NSCs with the bFGF/BMP4 combination induces quiescent neural stem cells (qNSCs). In this study, we performed bulk RNA sequencing (RNA-Seq) to compare the transcriptome profiles of BMP4-treated NSCs and bFGF/BMP4-treated NSCs, and found that both NSCs treated by these two methods were Sox2 positive qNSCs which were able to generate neurospheres. However, NSCs treated by those two methods exhibited different characteristics in state and the potential for neuronal differentiation based on transcriptome analysis and experimental results. We found that BMP4-treated NSCs tended to be in a deeper quiescent state than bFGF/BMP4-treated NSCs as the percentage of ki67-positive cells were lower in BMP4-treated NSCs. And after exposure to differentiated environment, bFGF/BMP4-treated NSCs generated more DCX-positive immature neurons and MAP2-positive neurons than BMP4-treated NSCs. Our study characterized qNSCs treated with BMP4 alone and bFGF/BMP4 combination, providing a reference for the scientific use of BMP4 and bFGF/BMP4-induced qNSCs models.

## 1 Introduction

The subventricular zone (SVZ) and subgranular zone (SGZ) are two major neurogenic niches in the adult mammalian brain where NSCs exist. Most of NSCs are in quiescent state, characterized by cell cycle arrest, and can be activated when brain injury occurs (Llorens-Bobadilla et al., 2015; Urbán et al., 2019). However, the migration of NSCs to the injured region and their differentiation into neurons in harsh microenvironment are inefficient (Wang et al., 2022; He et al., 2023). Understanding the characteristic of quiescent neural stem cells (qNSCs), effectively regulating the activation and differentiation of endogenous qNSCs is expected to improve the efficiency of NSCs in repairing cerebral neuronal injury.

BMP signaling is considered a regulator of qNSCs in both SVZ and SGZ (Mira et al., 2010; Armenteros et al., 2018; Marqués-Torrejón et al., 2021). Marqués-Torrejón et al. (2021) suggested that BMP4 signaling induces a dormant, noncycling qNSCs, but more evidence is needed to support this finding. Many studies have induced NSCs to enter a quiescent state by culturing NSCs in media supplemented with BMP4 and bFGF (FGF2, fibroblast growth factor *β*), which is regarded as a model for studying qNSC activation *in vitro* (Martynoga et al., 2013; Beckervordersandforth et al., 2017; Marqués-Torrejón et al., 2021). It was previously thought that the cells produced by BMP4-treated NSCs were astrocytes based on the fact that NSCs treated with BMP4 alone express GFAP. However, GFAP is expressed not only in astrocyte, but also in qNSCs (Jackson et al., 2006; Beckervordersandforth et al., 2014). Therefore, the characteristics of NSCs treated with BMP4 alone need further investigation. Comparing the transcriptome of BMP4- and bFGF/BMP4-treated NSCs with those of qNSCs and astrocytes *in vivo* can definitively determine whether BMP4-treated NSCs are qNSCs or astrocytes. And the different characteristics between bFGF/BMP4- and BMP4-treated NSCs need to be further studied, which help studying the mechanism of activation of qNSCs in different state.

In this study, we compared the transcriptome differences between NSCs treated with the bFGF/BMP4 combination and those treated with BMP4 alone, and identified the cell type of BMP4- and bFGF/BMP4-treated NSCs based on cell type-specific markers and cell type-specific gene expression matrix. After immunofluorescence validation, we determined that both NSCs treated with BMP4 alone and with bFGF/BMP4 are induced into qNSCs, but at different quiescent levels, with the former in a deep quiescent state and the latter in a shallow quiescent state. BMP4-treated NSCs in a deeper quiescent state are harder to be activated than bFGF/BMP4-treated NSCs, which are in a shallow quiescent state. NSCs treated with bFGF/BMP4 have better abilities to proliferate and differentiate into neurons. Our findings help researchers better understand the characteristics of qNSCs in different states induced by BMP4 and bFGF/BMP4, which will aid the study of the activation mechanism of qNSCs.

## 2 Materials and methods

### 2.1 Animals and cell culture

Wild-type adult mice (aged 8–9 weeks) were purchased from Shanghai SLAC Laboratory Animal Company and maintained at constant temperature and humidity with a 12-h light/dark cycle at Tongji University.

NSCs were isolated from the SVZs of adult C57BL/6 mice (2 female mice and 2 male mice were used for one cell isolation experiment) aged 8–9 weeks after euthanasia, which refer to the protocols described elsewhere (Walker and Kempermann, 2014; Leeman et al., 2018). Briefly, SVZs were microdissected and digested for 7 min at 37°C in EBSS (Gibco, 24,010,043) with 20 U/mL lyophilized papain (Worthing, LK003178), and then layered and spun over a 22% Percoll gradient (Cytiva, 17,089,102). And then the cells were maintained *in vitro* in proliferation medium, which was Dulbecco’s Modified Eagle Medium/Nutrient Mixture F-12 medium (DMEM/F12, Gibco, 11,330,032) supplemented with 1% penicillin streptomycin glutamine (P/S, Thermo, 10,378,016), 2% B27 minus vitamin A (Gibco, 12,587–010), and the growth factors EGF and bFGF (R&D Systems, 20 ng/mL each). NSCs obtained after at least three passages were plated on poly-L-ornithine (Sigma, P3655) and laminin (Sigma, L2020) at the density of 25,000 cells/cm^2^ and cultured in quiescence media [DMEM/F12, 2% B27, 1% P/S, 20 ng/mL bFGF (R&D Systems, 3,139-FB-025), and 20 ng/mL BMP4 (R&D Systems, 5,020-BP-010)] and BMP4-induced media [DMEM/F12, 2% B27, 1% P/S, and 20 ng/mL BMP4 (R&D systems)] for 6 days. For the activation of bFGF/BMP4-treated NSCs or BMP4-treated NSCs, the medium was changed to proliferation medium for another 6 days, and the cells were dissociated by Accutase (Gibco, A1110501) and then cultured in suspension.

For differentiation, the proliferation medium or BMP4-induced medium was changed to spontaneous differentiation medium (DMEM/F12, 2% B27, and 1% P/S) or neuron maintenance medium (Neurobasal™-A, 2 mM GlutaMAXTM-I, 2% B27, and 1% P/S).

The protocol of differentiation of ependymal cells from isolated NSCs was based on published methods (Paez-Gonzalez et al., 2011; Abdi et al., 2018; Omiya et al., 2021). Briefly, NSCs were cultured in Dulbecco’s modified Eagle’s medium containing high glucose (DMEM-High Glucose, KEL, KC302-01), and supplemented with 10% FBS (BI, 04-001-1ACS) and 1% P/S. After 5 days, the medium was switch to 2% FBS and continued to cultivate for 2 days.

### 2.2 GEO datasets

In this study, the normalized counts from GSE67833 were obtained from the NCBI GEO official website.^1^ The GSE67833 is a single-cell RNA sequencing data set. The normalized counts of 104 NSCs and 22 astrocytes of wild-type mice in GSE67833 were used in monocle and CIBERSORT analysis.

### 2.3 Bulk RNA-seq processing and DEG analysis

Total RNA was extracted from samples (1 × 10^6^ cells for one sample) using Trizol, and then the RNA qualification and qualification was determined by RNA Nano 6,000 Assay Kit of the Bioanalyzer 2,100 system (Agilent Technologies, CA, United States). RNA was prepared into cDNA and fragmented into 370 ~ 420 bp in length. The library fragments were purified with AMPure XP system (Beckman Coulter, Beverly, United States). Final libraries were prepared using TruSeq PE Cluster Kit v3-cBot-HS (Illumia) according to the manufacturer’s instructions, and them the library preparations were sequenced on an Illumina NovaSeq 6,000 platform (illumine, United States).

The quality of the raw reads in fastq format were investigated using fastp (version 0.19.7). Paired-end clean reads were aligned to the mm39 genome using HISAT2 v2.0.5. Differential expression analysis of the two groups was performed using the DESeq2 R package (version 1.32.0). The resulting *p* values were adjusted using the Benjamini and Hochberg’s approach for controlling the false discovery rate. Genes with an adjusted p value≤0.05 and logFC≥1 (FC ≥ 2) identified by DESeq2 were considered differentially expressed. Gene Ontology (GO) analysis was performed using the clusterProfiler R package (version 4.6.2).

### 2.4 Microarray significant profiles (maSigPro) analysis

We assumed a time line from bFGF/EGF-treated NSCs to bFGF/BMP4-treated NSCs and then to BMP4-treated NSCs to analyze the time-dependent genes using the maSigPro R package (version 1.66.0). Normalized FPKM data were used for single-series time course subjected to maSigPro analysis. The default parameters and quadratic regression model were used to identify temporally significant genes showing significant expression differences from the starting time (FDR ≤ 0.05). The temporally significant genes were then filtered by the second regression model, with the R^2^ parameter set to 0.6 and the vars parameter set to each. The significant genes were then grouped into five groups.

### 2.5 Analysis of potential cell components of bulk RNA-seq using single-sample GSEA (ssGSEA)

ssGSEA uses a rank-based method to identify a score representing the degree of the enrichment of a special gene set in each sample. The levels of different cell type populations were determined by ssGSEA using R package Gene Set Variation Analysis (GSVA, version 1.40.1) with the default parameters. The signature markers from published studies were used to generate gene lists for ssGSEA analysis (Supplementary Table S1) (Friedmann-Morvinski et al., 2016; Kleiderman et al., 2016; Shah et al., 2018; Mizrak et al., 2019).

### 2.6 Analysis of potential cell components of bulk RNA-seq using CIBERSORT

CIBERSORT uses the gene expression profile of different cell types from single-cell RNA sequencing to estimate the cell type composition of bulk RNA-sequencing data (Newman et al., 2015). We used a single-cell RNA sequence dataset to generate expression profiles for different types of cell types. In GSE67833, the 104 GLAST^+^Prom1^+^ cells isolated from the SVZs were regarded as NSCs. Five striatum astrocytes and 17 cortex astrocytes were GLAST^+^CD45^−^O4^−^. We distinguish aNSCs, qNSCs, and oligodendrocytes by a hierarchical clustering analysis that can exclude the 12 oligodendrocytes from NSCs isolated from wild-type mice according to the original paper (Supplementary Figure S1). NSC cluster associated with the expression of *egfr*, and genes associated with cell cycle (e.g., *Mki67* and *Mcm2*) were named aNSCs, and the cluster that lacked or have low expression levels of activation markers was named then qNSCs which had two types (qNSC1 and qNSC2) as description in the original paper (Llorens-Bobadilla et al., 2015). We further performed clustering, pseudotime, and expression difference analyses of the 92 NSCs and 22 astrocytes using the monocle R package (version 2.26.0). Because two astrocytes were included in qNSC2 cluster and one qNSC was included in astrocyte cluster, we excluded the data of these three cells in the follow analysis (Supplementary Figure S1B). The average normalized counts (FPKM) of 3,250 differentially expressed genes (*q* value<0.05, Supplementary Table S2) in every cell type was calculated to generated cell-type special gene expression matrix which was used in CIBERSORT performed in R.

### 2.7 Immunofluorescence

The cells were cultured on coverslips, fixed with 4% paraformaldehyde in PBS for 15 min, and then permeabilized with 0.2% Triton X-100 for 15 min. The cells were blocked in 3% BSA for 1 h at room temperature, incubated with primary antibodies at 4°C overnight and then incubated with secondary antibodies and DAPI (564,907, BD Pharmingen, 1:1000) at room temperature for 1 h. Coverslips were sealed with ProLongTM Diamond (Invitrogen, P36970). Images were captured by a confocal microscope (Leica SP8). The following primary antibodies were used: ARL13B (Proteintech, 17,711-1-AP, 1:400, anti-rabbit), acetyl-α-tubulin (Sigma, T6793, 1:500, anti-mouse), GFAP (Gene Tex, GTX85454, 1:400, anti-chicken), MAP2 (CST, #8707T, 1:200, anti-rabbit), DCX (Abcam, ab18723, 1:400, anti-rabbit), β-catenin (CST, #8480S, 1:200, anti-rabbit) and ki67 (ab15580, Abcam, 1:300, anti-rabbit), Nestin (ab6142, Abcam, 1:100, anti-mouse), Sox2 (Abclonal, A0561, 1:200, anti-rabbit), Donkey anti-Rabbit Alexa Fluor^™^488 (Invitrogen, A-21206, 1:1000), Donkey anti-Mouse Alexa Fluor^™^568 (Invitrogen, A10037, 1:1000), Donkey anti-Rabbit Alexa Fluor^™^555 (Invitrogen, A-31572, 1:1000), Goat anti-Chicken Alexa Fluor™647 (Invitrogen, A-21449, 1:1000), Donkey anti-Mouse Alexa Fluor^™^488 (Invitrogen, A-21202, 1:1000). And TUNEL (Vazyme, A113-03) was used to detected the apoptotic cell.

### 2.8 RT-qPCR

Total RNA was extracted from cells using RNAiso Plus (Takara, 9,109) following the manufacturer’s instructions. Reverse transcription was performed with HiScript III RT SuperMix for qPCR (Vazyme, R323-01). Quantitative real-time PCRs were conducted out using AceQ Universal SYBR qPCR Master Mix (Vazyme, Q511-02) and gene-specific primers (*Foxj1*-F: 5′-CTCCTATGCCACTCTCATCTGC-3′, *Foxj1*-R: 5′-GACAGGTTGTGGCGGATGGAAT-3′; *GAPDH*-F: 5′-CAGGTTGTCTCCTGCGACTT-3′, *GAPDH*-R: 5’-CCCTGTTGCTGTAGCCGTAT-3′). Relative expression levels of mRNA were calculated using the 2^-△△CT^ method normalized by the *GAPDH* level and compared with the control samples.

### 2.9 Statistical analysis

GraphPad Prism 8.0 (Graph Pad Software Inc., La Jolla, CA, United States) was used for the graphical representation and statistical analyses of the data. All the data are presented as the means ± SEMs, and each experiment was replicated at least three times. A two-tailed paired t test or one-way ANOVA with Tukey’s multiple comparison were used for the statistical analysis. The significance of the differences was assessed and indicated as follows: ns *p* > = 0.05, ∗ *p* < 0.05, ∗∗ *p* < 0.01, and ∗∗∗ *p* < 0.005 and ∗∗∗ *p* < 0.001.

## 3 Results

### 3.1 BMP4- and bFGF/BMP4-treated NSCs are qNSCs in different states, rather than astrocytes

**Figure 1:**
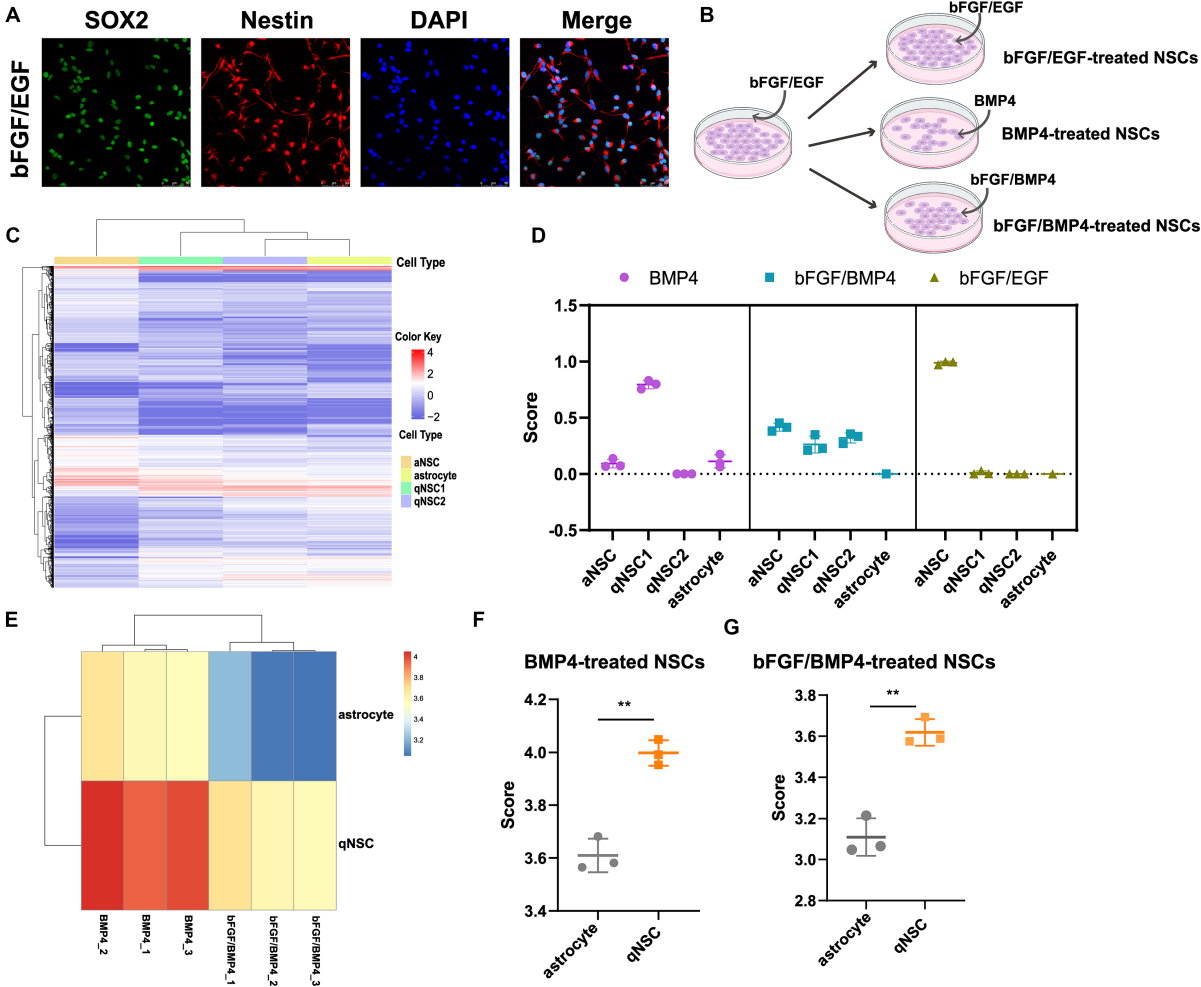
Identifying the cell type of BMP4-treated NSCs, bFGF/BMP4-treated NSCs and bFGF/EGF-treated NSCs by CIBERSORT and ssGSEA. **(A)** Immunofluorescence staining of Sox2 and Nestin in bFGF/EGF-treated NSCs. Scale bar: 50 μm. **(B)** Diagram of cell culture. **(C)** Heatmap of 3,250 DEGs, selected by monocle, among aNSCs, qNSCs and astrocytes in GSE67833. Cells are shown in columns, and genes are shown in rows. **(D)** The scores from the CIBERSORT analysis show the percentages of aNSC, qNSC1, qNSC2 and astrocyte among bFGF/EGF-treated NSCs, bFGF/BMP4-treated NSCs and BMP4-treated NSCs (*n* = 3/group). **(E)** Heatmap of the landscape of two gene sets in bFGF/BMP4-treated NSCs and BMP4-treated NSCs. Astrocyte is a gene set for astrocyte. qNSC is a gene set for qNSCs. **(F,G)** Histogram of the results in **(E)**. The data are presented as the means ± SEMs (two-tailed Student’s t-test), **p < 0.01.

In order to clarify the effect of BMP4 on NSCs, we performed bulk RNA-seq to compare the gene expression profiles of NSCs cultured in BMP4-induced medium and bFGF/BMP4-induced medium, and the gene expression profiles of NSCs cultured in bFGF/EGF-induced medium were used as activated NSCs (aNSC) control. Briefly, adult NSCs isolated from the mouse SVZs proliferated for several passages in proliferation medium supplemented with bFGF and EGF, and all the cells expressed Nestin and Sox2, two markers of NSCs, indicating that the bFGF/EGF-treated cells were pure NSCs (Figure 1A). Then we replaced bFGF and EGF in the medium with BMP4 as BMP4-treated NSCs, and replaced EGF with BMP4 as bFGF/BMP4-treated NSCs (Figure 1B). After 6 days of culture, the treated cells were collected for bulk RNA-seq.

The cell types of NSCs treated with BMP4 is controversial, and identifying the cell types of both BMP4- and bFGF/BMP4-treated NSCs will contribute to better analysis of their characteristics. Next, to identify the cell type of BMP4-, bFGF/BMP4- and bFGF/EGF-treated NSCs, we first used monocle to cluster single-cell RNA sequencing data GSE67833 and selected the significantly differentially expressed genes (DEGs, q value<0.05) in each cell type to construct a cell type-specific expression matrix that can distinguish aNSCs, qNSCs and astrocytes, and used it as a criterion for identifying cell types of BMP4-, bFGF/BMP4- and bFGF/EGF-treated NSCs (Supplementary Table S2; Figure 1C). We used CIBERSOT to calculate a score which reflects the hypothetical relative abundance score of aNSC, qNSC1, qNSC2 and astrocyte in BMP4-, bFGF/BMP4- and bFGF/EGF-treated NSCs, respectively (Figure 1D). The results showed that the abundance of qNSC in BMP4-treated NSCs was much higher than that of aNSC, and almost all bFGF/EGF-treated NSCs were aNSCs (Figure 1D). It is worth noting that the abundance of qNSC (qNSC1/qNSC2) in bFGF/BMP4-treated NSCs is close to that of aNSCs (Figure 1D), indicating that bFGF/BMP4-treated NSCs are in a shallow quiescent state, and qNSCs and aNSCs were in a dynamic equilibrium of mutual conversion.

To further identify BMP4- and bFGF/BMP4-treated NSCs as qNSCs and not astrocytes, we used ssGSEA to analyze the enrichment of qNSC and astrocyte marker gene sets in BMP4- and bFGF/BMP4-treated NSCs (Figures 1E–G). These results showed that the BMP4- and bFGF/BMP4-treated NSCs were enriched qNSC markers instead of astrocyte markers, further indicating that these cells are qNSCs rather than astrocytes.

### 3.2 BMP4-treated NSCs are qNSCs in a deeper quiescent state than bFGF/BMP4-treated NSCs

**Figure 2:**
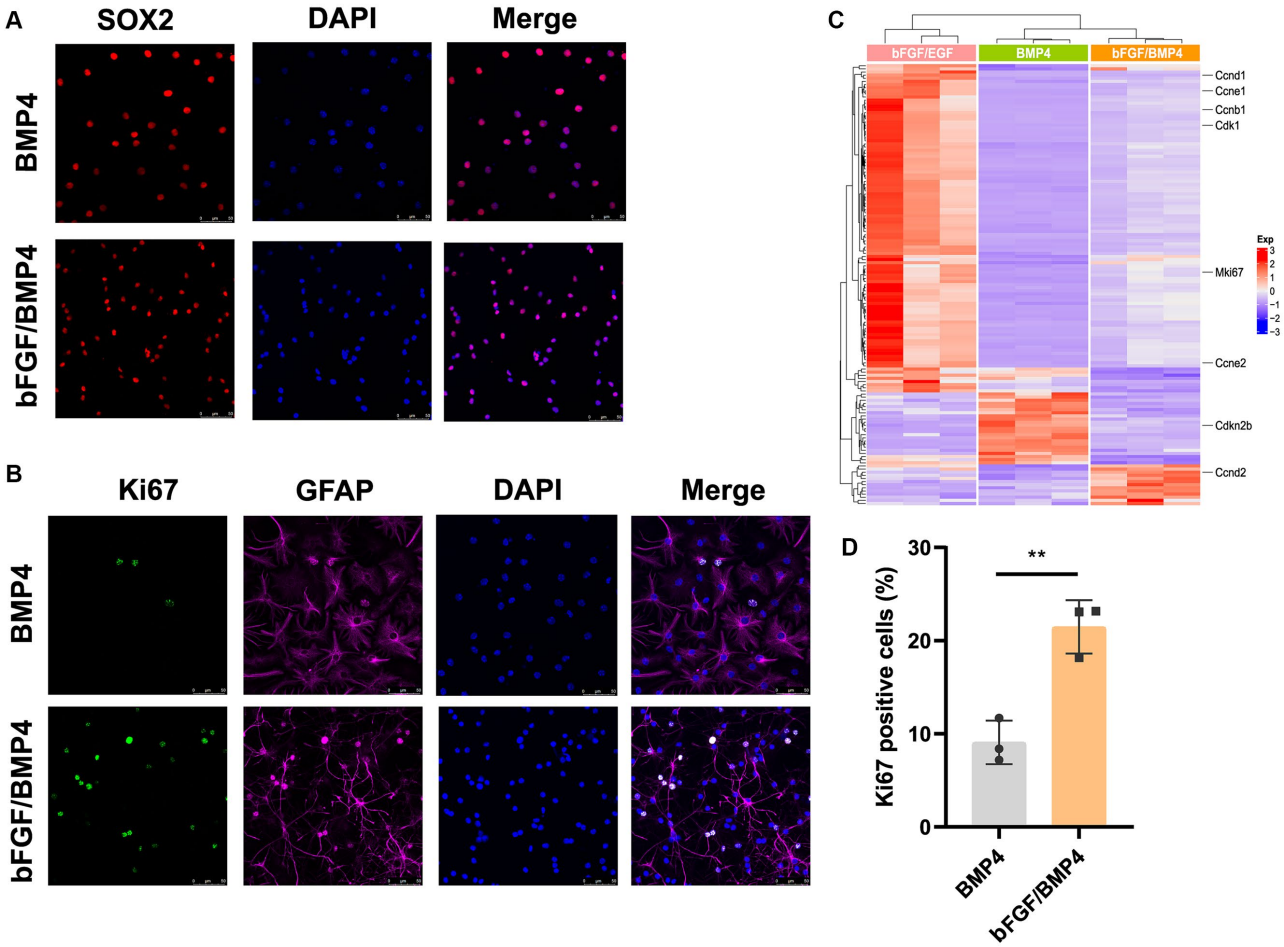
Detection of characteristics of qNSCs in BMP4- and bFGF/BMP4-treated NSCs. **(A)** Immunofluorescence staining of Sox2 in BMP4- and bFGF/BMP4-treated NSCs. Scale bar: 50 μm. **(B)** Immunofluorescence staining of ki67 in BMP4- and bFGF/BMP4-treated NSCs. Scale bar: 50 μm. **(C)** Heatmap of the expression levels of genes related to the cell cycle or chromosome segregation in bFGF/EGF-, bFGF/BMP4- and BMP4-treated NSCs. **(D)** Quantification of ki67-positive cells in **(B)** (*n* = 3/group). The data are presented means ± SEMs (two-tailed Student’s t-test), **p < 0.01.

**Figure 3:**
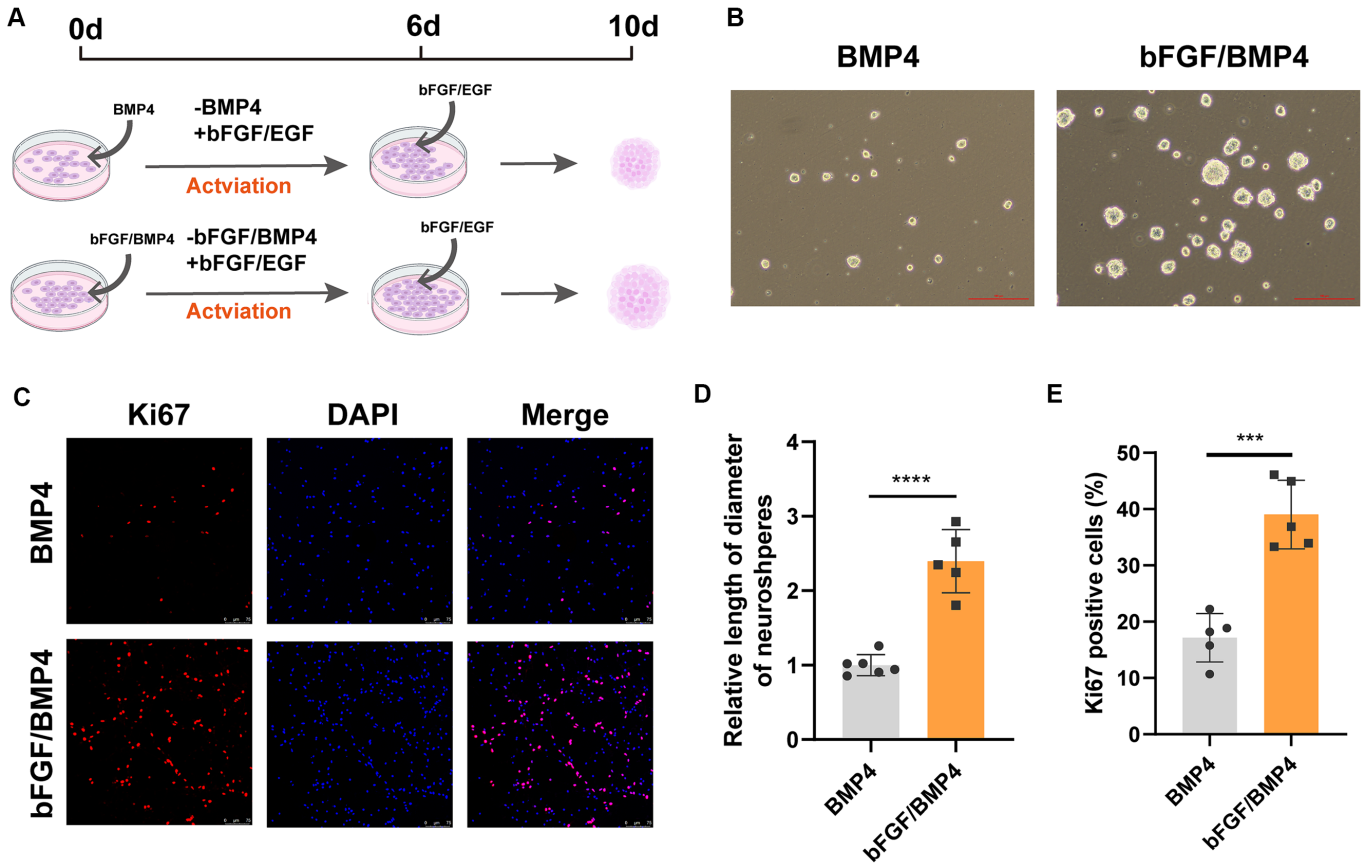
BMP4- and bFGF/BMP4-treated NSCs can be activated and re-enter into cell cycle. **(A)** Diagram of the activation of BMP4-treated NSCs and bFGF/BMP4-treated NSCs. To activated cells, the BMP4 was replaced by bFGF/EGF in BMP4-treated NSCs and the BMP4 was replaced by EGF in bFGF/BMP4-treated NSCs. Those cells were cultured in bFGF/EGF medium for 6 days, and then the cells were dissociated and cultured in suspension for 4 days. **(B)** Pictures of the neurospheres formed by BMP4- and bFGF/BMP4-treated NSCs after being activated at day 10 in **(A)**. Scale bar: 100 μm. **(C)** Immunofluorescence staining of ki67 in BMP4- and bFGF/BMP4-treated NSCs after being activated at day 6 in **(A)**. Scale bar: 75 μm. **(D)** Quantification of the diameter of neuroshperes in **(B)** (*n* = 5-6/group) in **(B)**. **(E)** Quantification of ki67-positive cells in **(C)** (*n* = 5/group). The data are presented means ± SEMs (two-tailed Student’s t-test), ***p < 0.005,****p < 0.001.

To further characterize BMP4- and bFGF/BMP4-treated NSCs, we investigated their levels of quiescence. We found that almost BMP4- and bFGF/BMP4-treated NSCs expressed Sox2 by performed immunofluorescence staining (Figure 2A). The proportion of ki67-positive cells in bFGF/BMP4-treated NSCs was higher than BMP4-treated NSCs (Figures 2B,D). We then compared the expression levels of genes associated with the cell cycle or chromatin segregation among NSCs treated BMP4 and bFGF/BMP4 by DESeq2, and found that the expression levels of genes that positively regulate the cell cycle, such as *Mki67*, *ccnd1*, *ccne1*, and *cdk1*, were higher in bFGF/EGF-treated NSCs than in BMP4-treated NSCs (Figure 2C; Supplementary Table S3). In contrast, *cdkn2b*, an encoded cyclin-dependent kinase inhibitor, was upregulated in BMP4-treated NSCs compared with NSCs subjected to bFGF/BMP4 (Figure 2C). In addition, it has been reported that qNSCs can be activated by growth factors such as EGF and VEGF and then enter the cell cycle to proliferate and differentiate (Doetsch et al., 2002; Luo et al., 2015). To compare the proliferation capacity of BMP4- and bFGF/BMP4-treated NSCs, we activated them by EGF and then cultured these cells in suspension and in proliferation medium (bFGF/EGF). We found that bFGF/BMP4-treated NSCs produced larger neurospheres (Figures 3A,B,D) and that the proportion of ki67-positive cells in bFGF/BMP4-treated NSCs was higher than that in BMP4-treated NSCs after being activated (Figures 3C,E), indicating that BMP4-treated NSCs are in a deep quiescent state while bFGF/BMP4-treated NSCs are in a shallow quiescent state that is more easily activated.

### 3.3 The differences of transcriptome characteristics between BMP4- and bFGF/BMP4-treated NSCs

**Figure 4:**
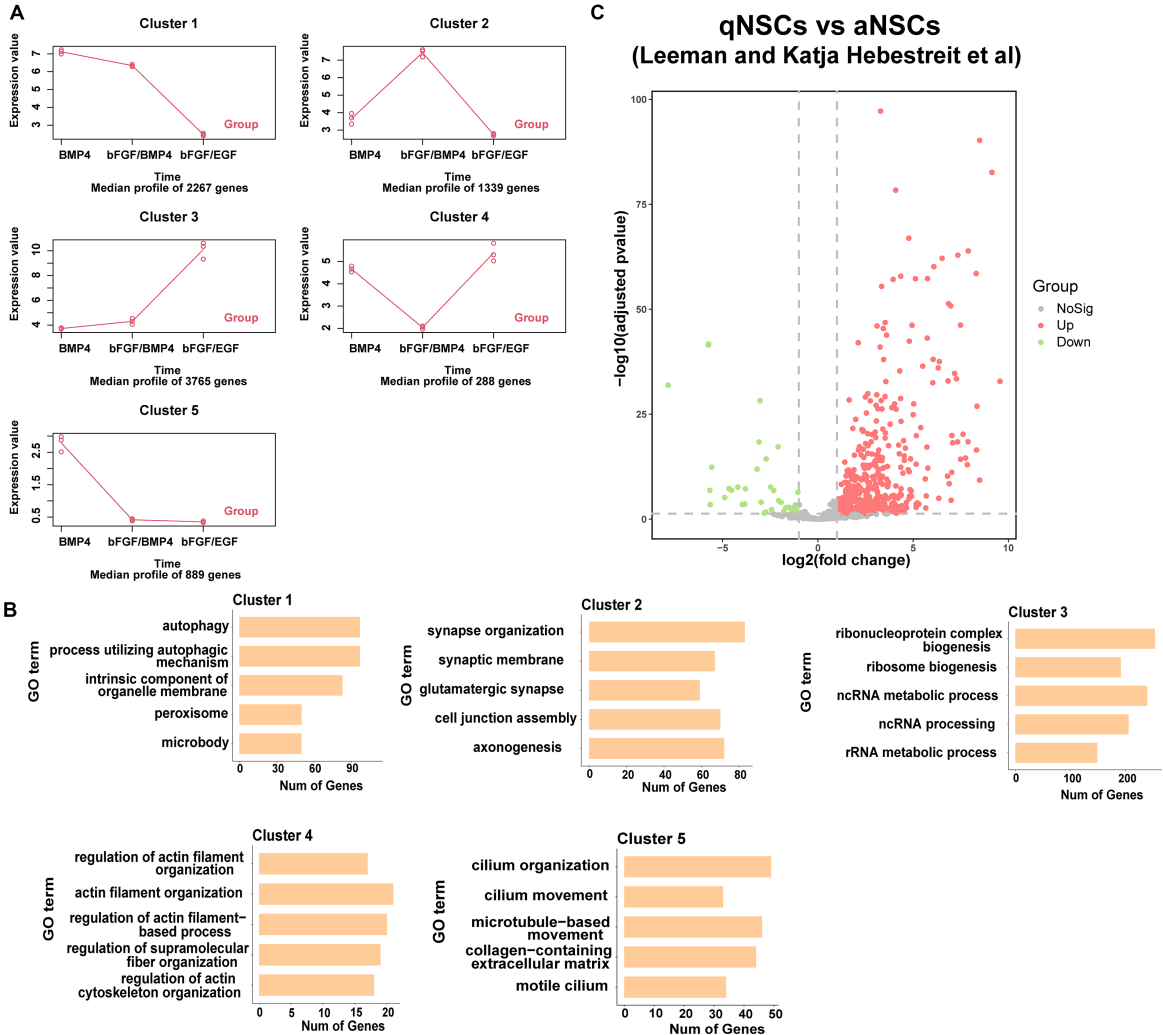
Functional enrichment of differentially expressed genes between BMP4-treated NSCs and bFGF/BMP4-treated NSCs. **(A)** maSigPro results showing the main gene expression profile of each cluster. **(B)** GO analysis of the genes in each cluster in **(A)**. **(C)** Volcano plots showing the differential expression of cluster 2 genes classified by maSigPro in primary aNSCs and qNSCs isolated from SVZs of adult mice in published article.

To found the differences of transcriptome characteristics between BMP4- and bFGF/BMP4-treated NSCs, we hypothesized that bFGF/BMP4-treated NSCs are in an intermediate state between BMP4-treated NSCs and bFGF/EGF-treated NSCs, and performed a time-series analysis with maSigPro. The results showed that 8,548 genes exhibited pseudotemporal changes, and were classified into 5 clusters with different expression profiles (Figure 4A). Specifically, the expression of 3,765 genes related to cell proliferation in cluster 3 increased gradually from BMP4 to bFGF/BMP4 treatment, whereas the expression of 2,267 genes in cluster 1 related to autophagy and peroxisomes decreased from BMP4 to bFGF/BMP4 treatment (Figures 4A,B). The 1,339 genes in cluster 2 were uniquely upregulated by bFGF/BMP4 treatment, and were prominently enriched in terms or pathways such as synapse organization, synaptic membrane, GTPase activator activity, and glutamatergic synapse (Figures 4A,B). We compared the expression levels of the 1,339 genes between qNSCs and aNSCs which are isolated from the SVZs of adult mice reported by fluorescence-activated cell sorting (FACS) (Leeman et al., 2018). The results showed that 442 out of 1,339 genes were upregulated and 39 out of 1,339 genes were downregulated in qNSCs compared with aNSCs (Figure 4C), which indicated that most genes in cluster 2 related to synapses and axons were upregulated in qNSCs compared with aNSCs under physiological conditions. The 288 genes in cluster 4 associated with actin filament organization and cytoskeleton organization were uniquely downregulated in bFGF/BMP4-treated NSCs, which showed that there may be different between BMP4- and bFGF/BMP4-treated NSCs in cell shape, motility and interaction with environment (Figures 4A,B). On the other hand, the 889 genes in cluster 5, which were highly expressed in BMP4-treated NSCs, were associated with cilium organization and cilium movement (Figures 4A,B), which showed that BMP4-treated NCSs have high ciliogenesis levels.

Taken together, these results indicated that BMP4- and bFGF/BMP4-treated NSCs exhibited different developmental states and different characteristics. bFGF/BMP4-treated NSCs typically upregulated genes associated with the development of synapses, whereas BMP4-treated NSCs typically upregulated genes associated with the development of cilia.

### 3.4 BMP4-treated NSCs had more developed cilia than bFGF/BMP4-treated NSCs

**Figure 5:**
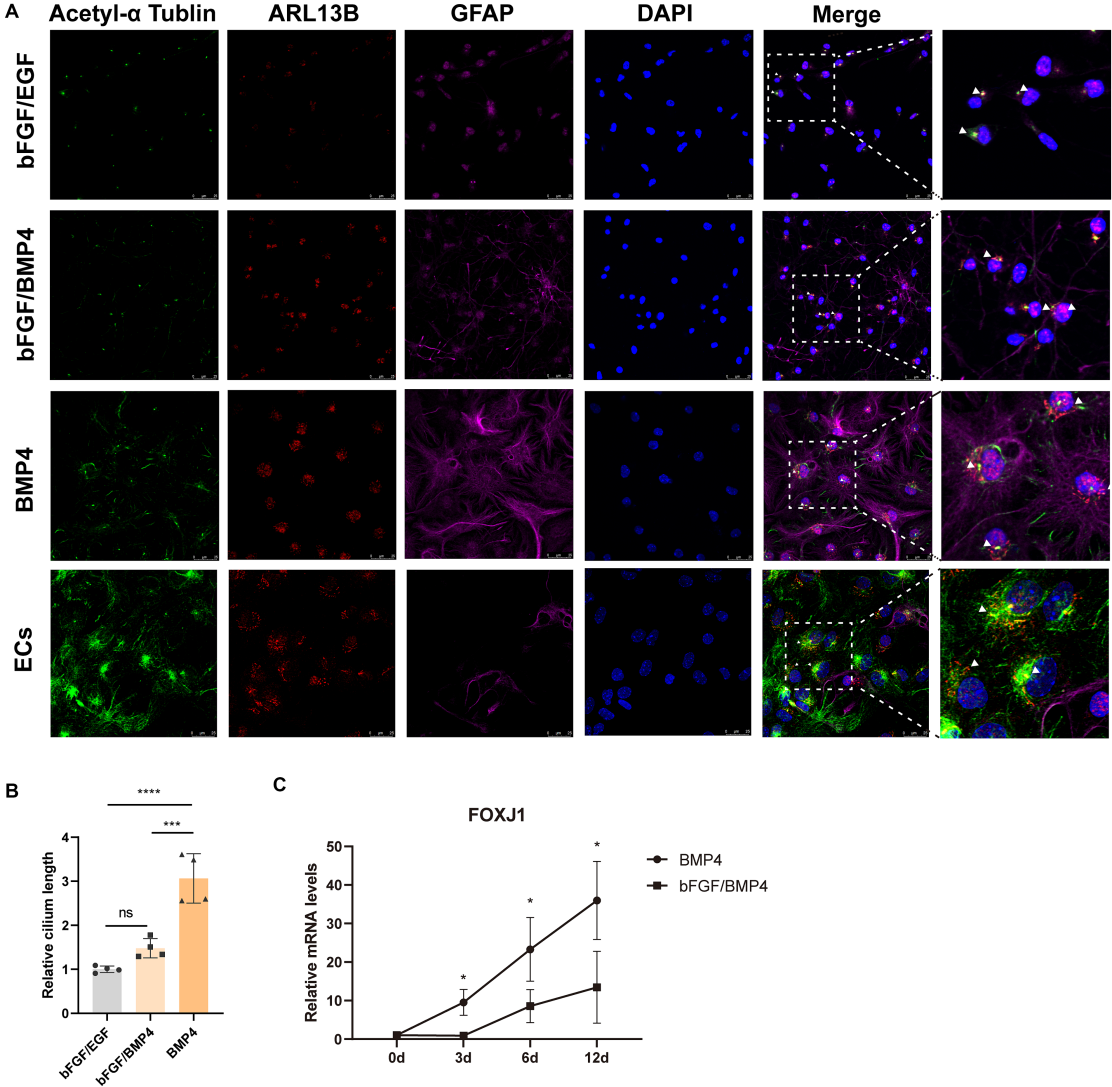
Analysis of the cilia characteristics of BMP4-treated NSCs, bFGF/BMP4-treated NSCs, bFGF/EGF-treated NSCs, and NSCs-induced ependymal cells (ECs). **(A)** Immunofluorescence staining of acetyl-α tubulin, ARL13B and GFAP. Scale bar: 25 μm. **(B)** Quantification of the relative cilium length in **(A)**. The cilium which marked both ARL13B and acetyl-α tubulin was measured in image J (*n* = 4/group). We set the longest value of the cilia from one end to the other as its length. The triangular arrow indicates the typical cilia. The data are presented as the means ± SEMs (one-way ANOVA with Tukey’s multiple comparison), ns *p* > =0.05, *** *p* < 0.005, **** *p* < 0.001. **(C)** The variation trend of mRNA expression level of *Foxj1* during bFGF/BMP4 and BMP4 treatment (*n* = 3/group). The data are presented as the means ± SEMs (two-tailed Student’s *t*-test), *p < 0.05.

The result from gene enrichment analysis of maSigPro revealed that the genes in cluster 5 enriched in the GO terms of cilium movement and motile cilium were upregulated in BMP4-treated NSCs (Figures 4A,B). We then compared the expression levels of a catalog of genes encoding for proteins related to either motile cilia (88 genes) or related to primary cilia (74 genes) reported by Olesia lgnatenko (Supplementary Table S4; Supplementary Figure S2) (Ignatenko et al., 2023). The expression of 48 out of 88 motile cilia marker genes and 31 out of 74 primary cilia marker genes differed between BMP4- and bFGF/BMP4-treated NSCs, and the majority of differentially expressed genes were upregulated in BMP4-treated NSCs (Supplementary Figure S2). *Foxj1* (Forkhead Box J1), a key regulator of motile ciliogenesis, was significantly upregulated in BMP4-treated NSCs compared with bFGF/BMP4-treated NSCs, and the expression level of *Foxj1* increased with extension of BMP4 treatment time (Figure 5C), indicating BMP4 alone treatment of NSCs could increase the ciliogenic development of NSCs than those treated with bFGF/BMP4. Long motile cilium is a characteristic of ependymal cells (ECs), and NSCs has short primary cilium. To confirm whether the cilia of BMP4-treated NSCs were similar with those of ECs, we induced NSCs into ECs based on published methods and identified the cilia with ARL13B and acetyl-tubulin (Abdi et al., 2018; Omiya et al., 2021). ECs have a large apical surface and long motive cilia (Codega et al., 2014; Deng et al., 2023). We found that ECs induced from NSCs displayed the typical characteristics of ECs, which clustered closely arranged cilia (Supplementary Figure S3; Figure 5A). Although BMP4-treated NSCs had cilia on their surfaces, the cilia were sparsely distributed (Figure 5A). Moreover, the cilia of bFGF/EGF- and bFGF/BMP4- treated NSCs were shorter than those of NSCs treated with BMP4 alone (Figures 5A,B). In addition, the morphological structure of NSCs treated with BMP4 and bFGF/BMP4 and bFGF/EGF was different from that of ECs (Supplementary Figure S3). Therefore, BMP4 treatment of NSCs do not develop motile cilium like ECs, and the primary cilia in BMP4-treated NSCs are more developed than that in bFGF/BMP4-treated NSCs.

### 3.5 bFGF/BMP4-treated NSCs differentiate into neurons more easily than NSCs treated with BMP4 alone

**Figure 6:**
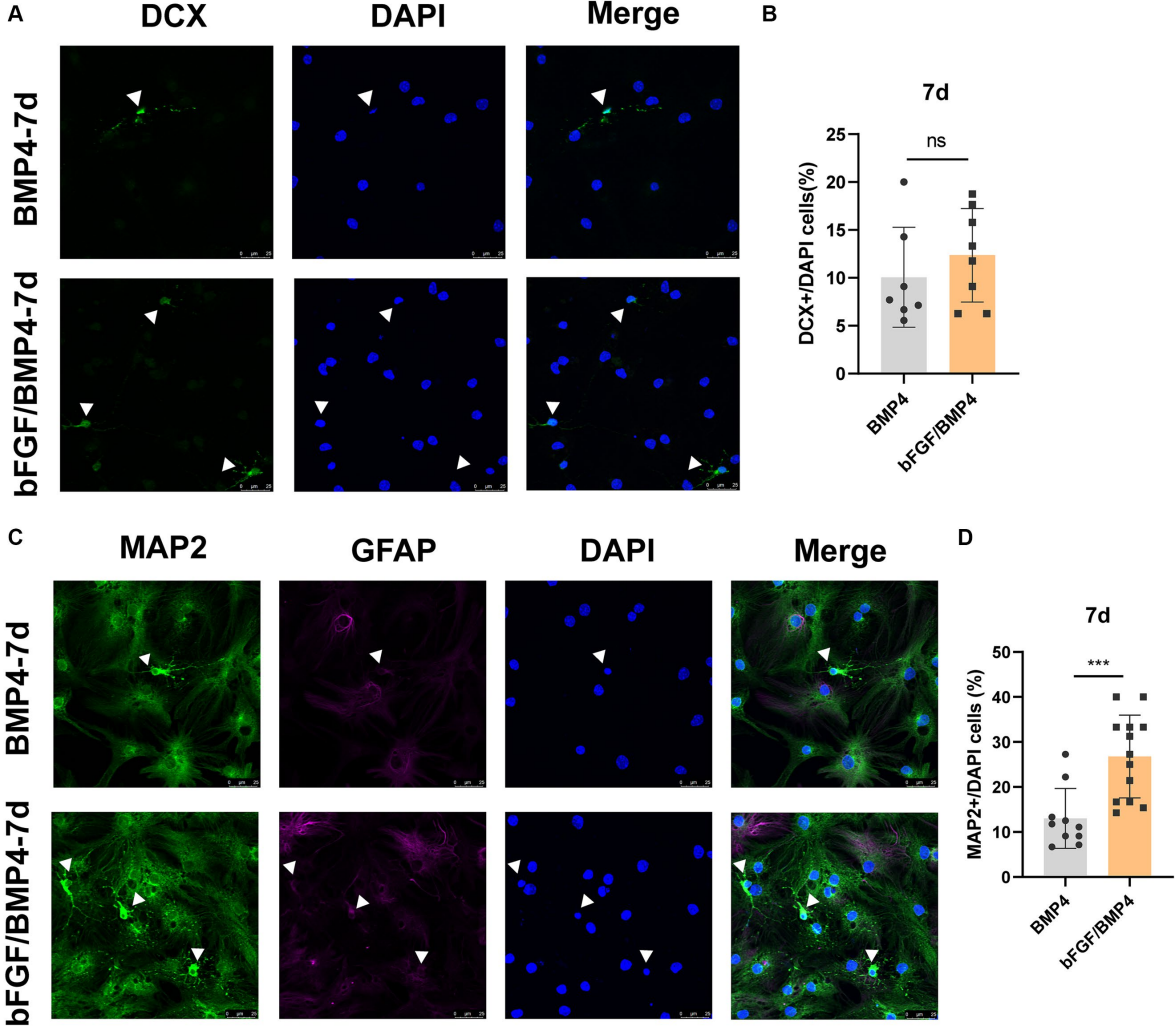
Immunofluorescence staining of MAP2^+^ and DCX^+^ neurons in BMP4-treated NSCs and bFGF/BMP4-treated NSCs after exposing to neuron-maintenance medium. After BMP4 or bFGF/BMP4 treatment for 6 days, the medium was changed to neuron-maintenance medium, and the cells were cultivated for 7 days. **(A)** Immunofluorescence staining of DCX^+^ cells at 7d after differentiation. Scale bar: 25 μm. **(B)** Percentages of DCX^+^ cells in **(A)** (*n* = 7-8/group). **(C)** Immunofluorescence staining of MAP2^+^GFAP^−^ cells at 7d after differentiation. Scale bar: 25 μm. **(D)** Percentages of MAP2^+^GFAP^−^ cells in **(C)** (*n* = 10-13/group). The data are presented as the means ± SEMs (two-tailed Student’s *t*-test), ns *p* > =0.05, *** *p* < 0.005.

To explore the neuron-induced differentiation potential of BMP4- and bFGF/BMP4-treated NSCs when they were in quiescent state, we used neuron maintenance medium (Neurobasal^™^, 2% B27, and 20 mM GlutaMAX^™^-I) to induce the differentiation of BMP4- and bFGF/BMP4-treated NSCs into neurons *in vitro*, with DCX as an immature neuron marker and MAP2 as a mature neuron marker. Both BMP4- and bFGF/BMP4-treated NSCs were able to develop into DCX^+^ neurons and MAP2^+^ neurons with neuronal morphology, and MAP2^+^ neurons exhibited weak GFAP expression (Figure 6). However, BMP4-treated NSCs developed into a lower proportion of MAP2^+^ neurons than that obtained with bFGF/BMP4-treated NSCs in neuron-maintenance medium.

**Figure 7:**
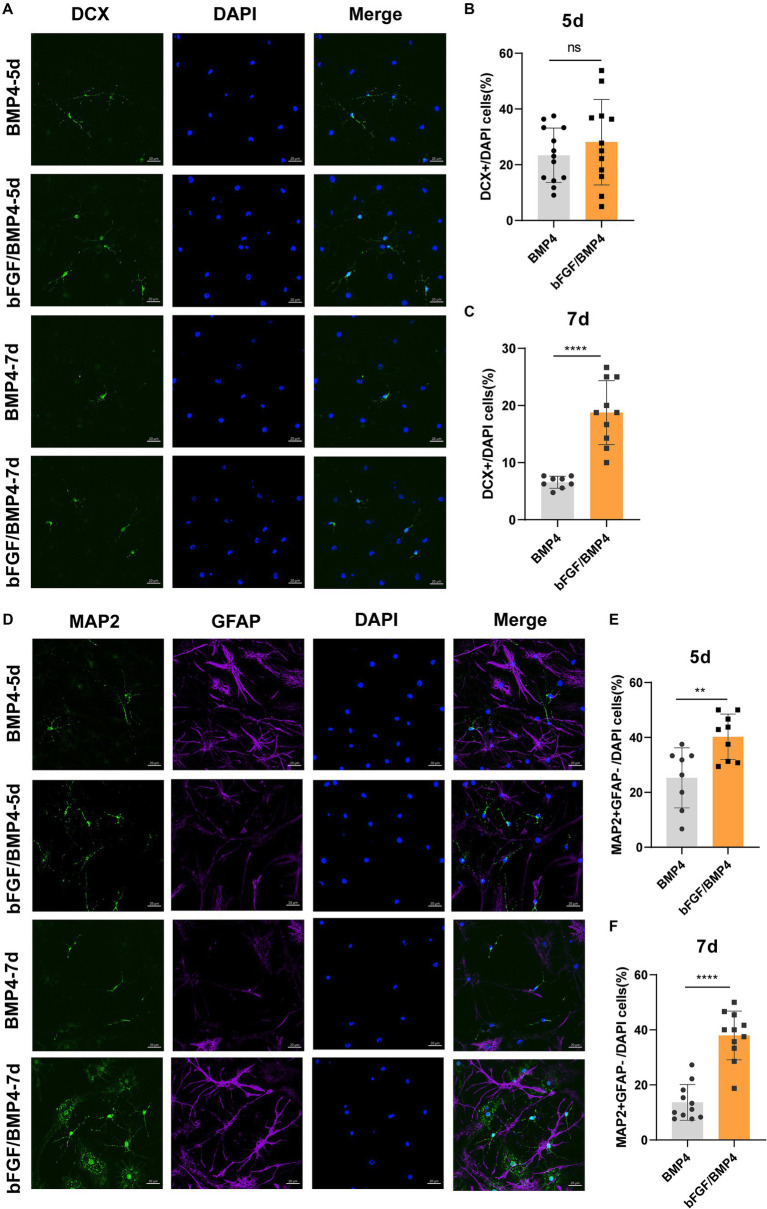
Detection of the ability of BMP4- or bFGF/BMP4-treated NSCs to differentiate into DCX^+^ and MAP2^+^ neurons after being activated. **(A)** Immunofluorescence staining of DCX in BMP4- and bFGF/BMP4-treated NSCs at 5d and 7d after differentiation. Scale bar: 20 μm. **(B)** Percentages of DCX^+^ cells at 5d after differentiation in **(A)** (*n* = 12–13/group). **(C)** Percentages of DCX^+^ cells at 7d after differentiation in **(A)** (*n* = 8–10/group). **(D)** Immunofluorescence staining of MAP2 and GFAP in BMP4- and bFGF/BMP4-treated NSCs at 5d and 7d after differentiation. Scale bar: 20 μm. **(E)** Percentages of MAP2^+^GFAP^−^ cells at 5d after differentiation in **(D)** (*n* = 8–9/group). **(F)** Percentages of MAP2^+^GFAP^−^ cells at 7d after differentiation in **(D)** (*n* = 11/group). The data are presented as the means ± SEMs (two-tailed Student’s *t*-test), ns *p* > =0.05, ** *p* < 0.01, **** *p* < 0.001.

To explore the potential of spontaneous differentiation of BMP4- and bFGF/BMP4-treated NSCs, we exposed the cells to spontaneous medium. However, we found that it is not easy to differentiate spontaneously in BMP4- and bFGF/BMP4-treated NSCs when they were in quiescent state. Then, we activated BMP4- and bFGF/BMP4-treated NSCs using EGF, cultured them in suspension, digested the neurospheres, and cultured them adherently in spontaneous medium to induce their differentiation. After 5d of differentiation, bFGF/BMP4-treated NSCs produced a similar proportion of DCX^+^ neurons but a higher proportion of MAP2^+^GFAP^−^ neurons compared with BMP4-treated NSCs, and after 7d, bFGF/BMP4-treated NSCs produced a higher proportion of DCX^+^ and MAP2^+^GFAP^−^ neurons (Figure 7). And in the BMP4-treated NSCs, the proportion of DCX^+^ and MAP2^+^ neurons decreased on the 7th day of differentiation compare to the 5th day, which might be due to apoptosis (Supplementary Figures S4, S5). This finding showed that bFGF/BMP4-treated NSCs differentiate into more MAP2^+^ mature neurons than BMP4-treated NSCs, indicating bFGF/BMP4-treated NSCs have stronger ability to differentiated into neurons upon activation.

Taken together, BMP4- and bFGF/BMP4-treated NSCs can be activated and differentiate into neurons, and bFGF/BMP4-treated NSCs differentiate into neurons more easily not only when they are exposed to neural maintenance medium but also when they are treated with spontaneous differentiation medium after being activated.

## 4 Discussion

To better highlight the differences between BMP4-treated NSCs and bFGF/BMP4-treated NSCs, we performed bulk RNA-seq of these two sets of NSCs and compared their transcriptome signatures. We found that both bFGF/BMP4-treated NSCs and BMP4-treated NSCs exhibited characteristics of qNSCs rather than astrocytes, and were in different states with different differentiation potentials. bFGF/BMP4-treated NSCs express synaptogenesis-related genes, whereas BMP4-treated NSCs are more quiescent and have higher expression levels of ciliogenesis-related genes. Moreover, bFGF/BMP4-treated NSCs are found to exhibit a stronger ability to differentiate into neurons than BMP4-treated NSCs.

Previous study suggested that BMP4 treatment of NSCs induced astrocytes *in vitro*, and GFAP is used as a marker of astrocytes (Gross et al., 1996; Shi et al., 2023). However, qNSCs share many markers with astrocytes, including GFAP (Dulken et al., 2017; Wang et al., 2021). Recently, Marqués-Torrejón et al. (2021) suggested that BMP4 induce a dormant qNSCs *in vitro* in the absent of FGF2, however only CD9 is used to distinguish the qNSCs from astrocytes. In this study, we established a cell type-specific expression matrix to distinguish qNSC1, qNSC2 and aNSC groups, rather than relying solely on a single or a few genes to distinguish these groups, thus effectively improving the accuracy of distinguishing these cell groups. We find that BMP4- and bFGF/BMP4-treated NSCs are qNSCs rather than astrocytes according to their transcriptomic signatures, and both of these cells express GFAP and Sox2, the two markers of qNSCs (Abdi et al., 2018; Urbán et al., 2019; Omiya et al., 2021). Sox2 is a marker for cells with stemness. BMP4 has been reported to rest mouse epiblast stem cells to a state of naïve pluripotency through ZBTB7A/B, which open the chromatin of specific genes, including Sox2, thereby permitting their expression (Yu et al., 2020). There may be a similar mechanism for maintain the expression of Sox2 in both bFGF/BMP4- and BMP4-treated NSCs, compared to the transition from primitive to naïve of mouse pluripotent stem cells, which is mediated by BMP4. Therefore, our findings further demonstrated that BMP4 treated NSCs alone induced qNSCs *in vitro*.

The apical end of type B1 cell (NSCs) and ECs are the center of pinwheel architecture to the ventricular surface of SVZ (Mirzadeh et al., 2008; Rodriguez-Jimenez et al., 2019). NSCs has one shorter primary cilium, but ECs has two or more longer cilia (Mirzadeh et al., 2008; Amador-Arjona et al., 2011). We find that the cilia of BMP4-treated NSCs were single and short primary cilia, similar to the cilia of bFGF/BMP4-treated NSCs and bFGF/EGF-treated NSCs, but different from the multiple cilia of ECs induced from NSCs. However, BMP4-treated NSCs have relatively longer primary cilia than bFGF/BMP4-treated NSCs, which is an important morphological marker for distinguishing these cells. The extension of cilium is happened in G1/G0 of cell cycle, and the disassembling of extended cilium means that cells enter into other cell cycle phase (Paridaen et al., 2013; Malicki and Johnson, 2017), so the length of the primary cilia is negatively correlated with cell cycle progression (Li et al., 2011; Wilson et al., 2012; Patel and Tsiokas, 2021). Therefore, compared to bFGF/BMP4-treated NSCs, the longer cilium of BMP4-treated NSCs correspond to the deeper state of quiescence. Moreover, the primary cilia of qNSCs are in close contact with the cerebrospinal fluid in lateral ventricles, which play a key role in the reception of extracellular signals to regulate adult neurogenesis(Lehtinen and Walsh, 2011; Falcão et al., 2012).

NSCs isolated form SGZ have the same ability to self-renew and differentiate into neurons as the same as NSCs isolated form SVZ (Ma et al., 2009). It is reported that BMP4 can also induced NSCs isolated from SGZ to be a quiescent state (Mira et al., 2010), and the primary cilia of qNSCs in SGZ are longer than those of aNSCs (Bhattarai, 2015). We presume that NSCs isolated from SGZ and treated with BMP4 may exhibit a more quiescent state compared to those treated with bFGF/BMP4, and that length of cilia may be associated with the quiescent state of NSCs in SGZ. However, more studies are required to prove this hypothesis, and the difference between NSCs from SVZ and SGZ treated with BMP4 should be taken further study.

Moreover, we found that genes associated with synapse development were upregulated in bFGF/BMP4-treated NSCs compared to BMP4-treated NSCs, and most of these genes were more highly expressed in qNSCs than in aNSCs, both isolated from the SVZs of mice using FACS. The gene associated with synaptogenesis are multifunctional and the development of synapse involves variety of pathways. For example, extracellular matrix and cell adhesion are reported to regulated both synapse development and maintaining stem cell quiescence (Sun and Xie, 2012; Shin et al., 2015; Morizur et al., 2018; Yang et al., 2023). The genes associated with synapse development are likely to exert a range of functions in qNSCs, and this possibility remains to be studied.

In summary, BMP4-treated NSCs are a subset of qNSCs *in vitro* rather than astrocytes, and bFGF/BMP4-treated NSCs are more easily be activated and induced to differentiate into neurons. This study clarified the characteristics of BMP4- and bFGF/BMP4-treated NSCs at molecular and cellular levels, which is helpful for further research on the mechanism of quiescent state maintenance and activation of qNSCs.

## Data availability statement

All data generated or analyzed during this study are included in this published article. The raw counts of bulk RNA-Seq datasets generated here are available in the Supplementary Table S5 of this article. The data presented in the study are deposited in the NCBI Gene Expression Omnibus (GEO) database repository, accession number GSE267115.

## Ethics statement

The animal study was approved by Tongji University (Project name: effect and mechanism of endogenous stem cells on injury repair of ischemic stroke, Date of approval: 24/8/2020, Project number: TJAA08820101). The study was conducted in accordance with the local legislation and institutional requirements.

## Author contributions

SX: Conceptualization, Methodology, Software, Writing – original draft, Writing – review & editing. XZ: Formal analysis, Validation, Writing – review & editing. ZL: Investigation, Writing – review & editing. CheL: Data curation, Writing – review & editing. QL: Resources, Writing – review & editing. HC: Investigation, Writing – review & editing. HY: Visualization, Writing – review & editing. YL: Writing – review & editing. SL: Funding acquisition, Supervision, Writing – review & editing. ChuL: Conceptualization, Project administration, Writing – review & editing.

## Footnotes

^1^ http://www.ncbi.nlm.nih.gov
