# Supplementary material for: Characteristics of quiescent adult neural stem cells induced by the bFGF/BMP4 combination or BMP4 alone in vitro
Source: Front Cell Neurosci. 2024 May 22;18:1391556. doi: 10.3389/fncel.2024.1391556 (PMC11151745; doi:10.3389/fncel.2024.1391556)
Supplement: Supplementary file 6 [file Data_Sheet_1.docx]

Supplementary Material

# Supplementary Figures and Tables

## Supplementary Figures


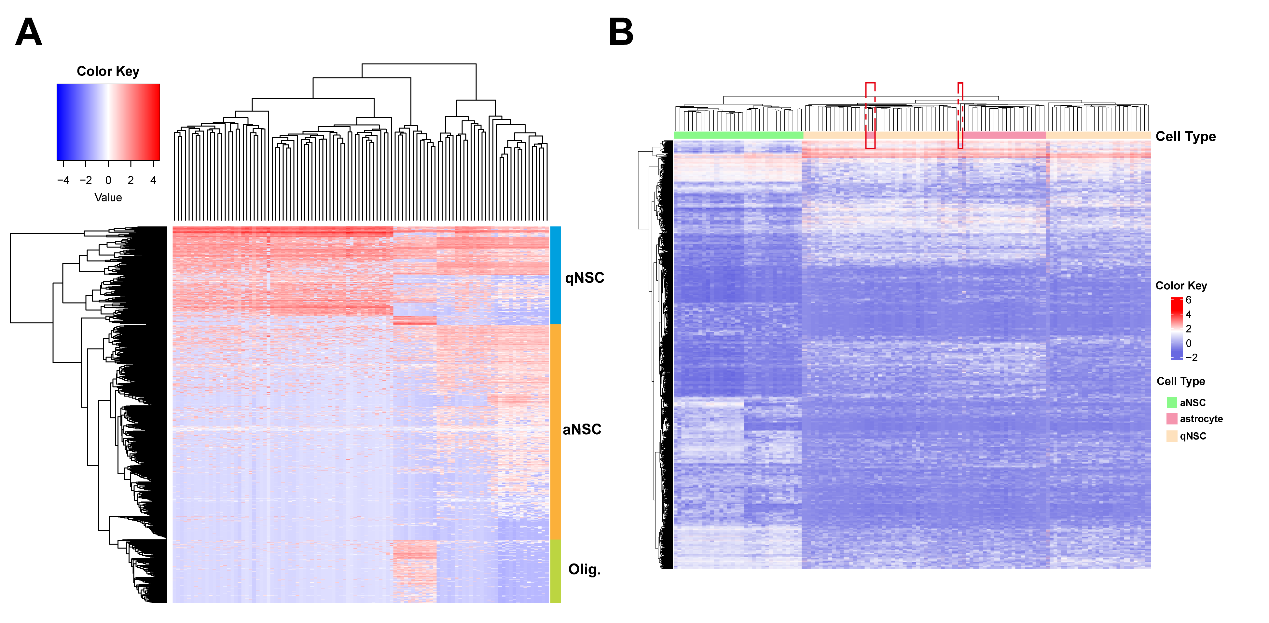


**Supplementary Figure S1**. Data preprocession of GSE67833. (**A**) Heatmap of 104 NSCs in GSE67833, which included 61 qNSCs, 31 aNSCs, and 12 oligodendrocytes. (**B**) Heatmap of 61 qNSCs, 31 aNSCs and 21 astrocytes using a significant genes matrix generated with monocle. One qNSC and two astrocytes indicated by the red dotted line were excluded to produce a special genetic matrix for CIBERSORT analysis.


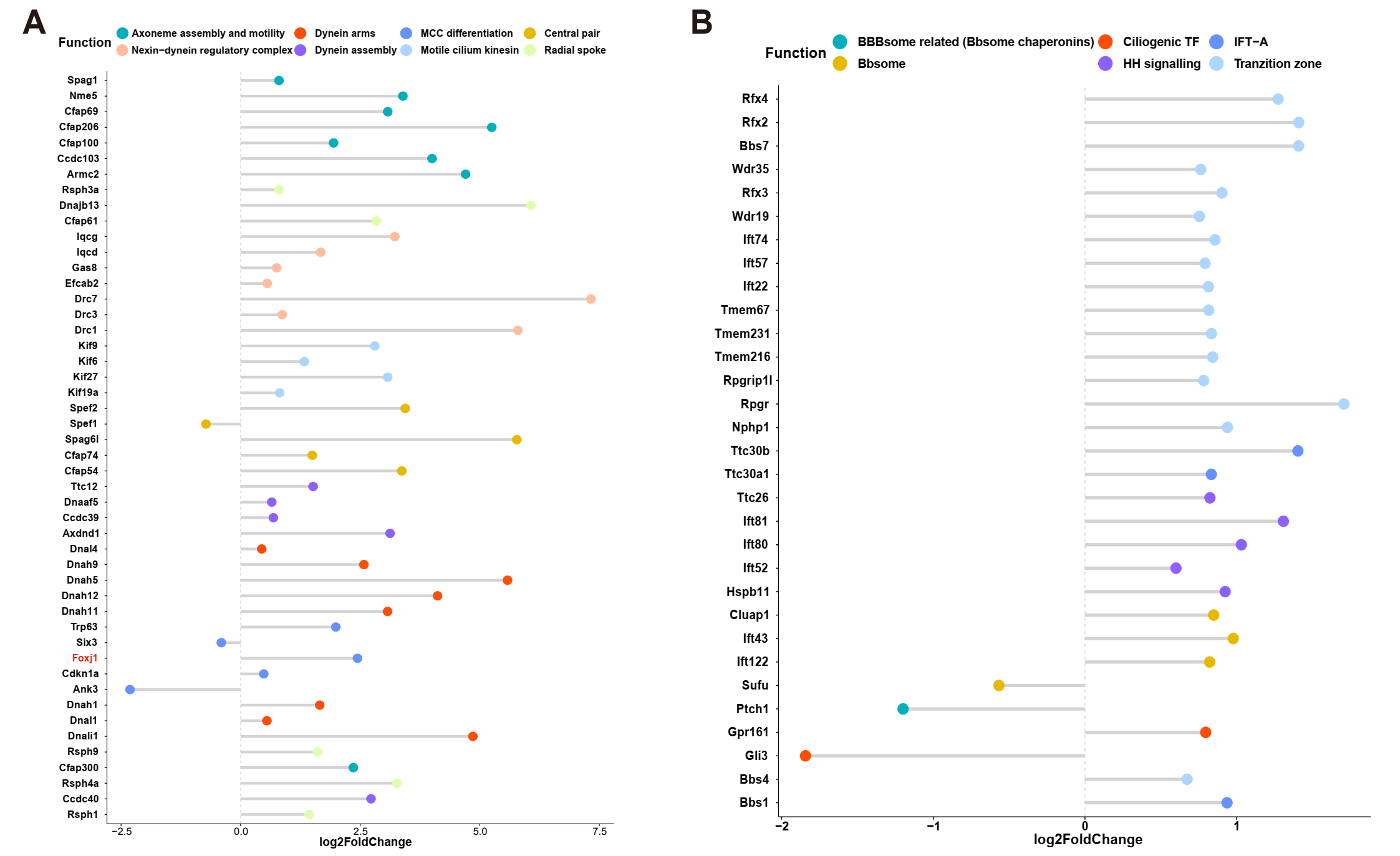


**Supplementary Figure S2.** Expression of specific DEGs involved in the development of motile cilia, primary cilia in BMP4-treated NSCs versus bFGF/BMP4-treated NSCs **(A)** The plot shows the expression levels of marker genes of motile cilia in BMP4-treated NSCs versus bFGF/BMP4-treated NSCs. **(B)** The plot shows the expression levels of marker genes of primary cilia in BMP4-treated NSCs versus bFGF/BMP4-treated NSCs.


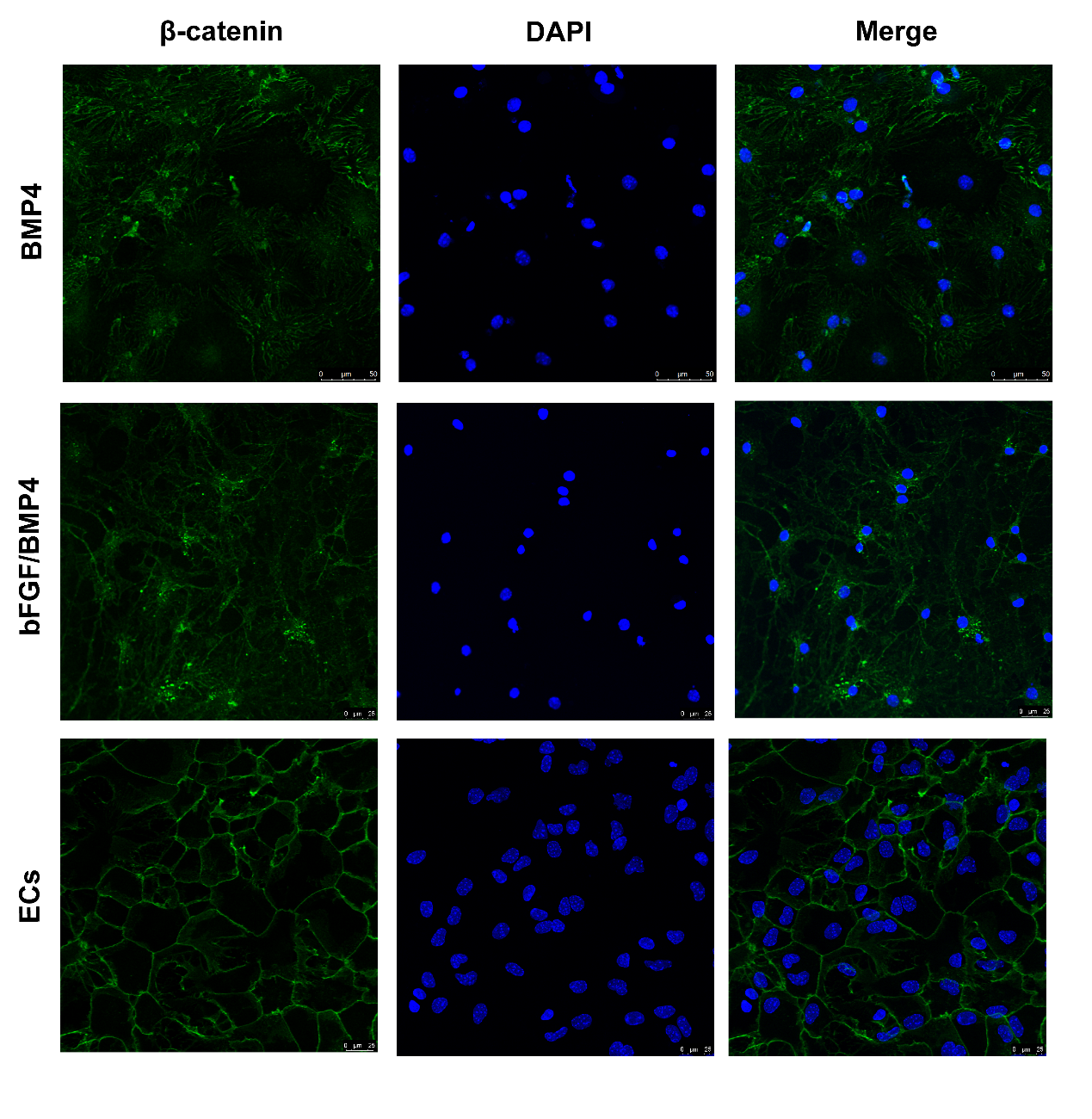


**Supplementary Figure S3.** Typical large apical surface of NSCs-ependymal cells. Immunofluorescence staining for β-catenin was performed to outline the cells morphology. Scale bar: 25μm.


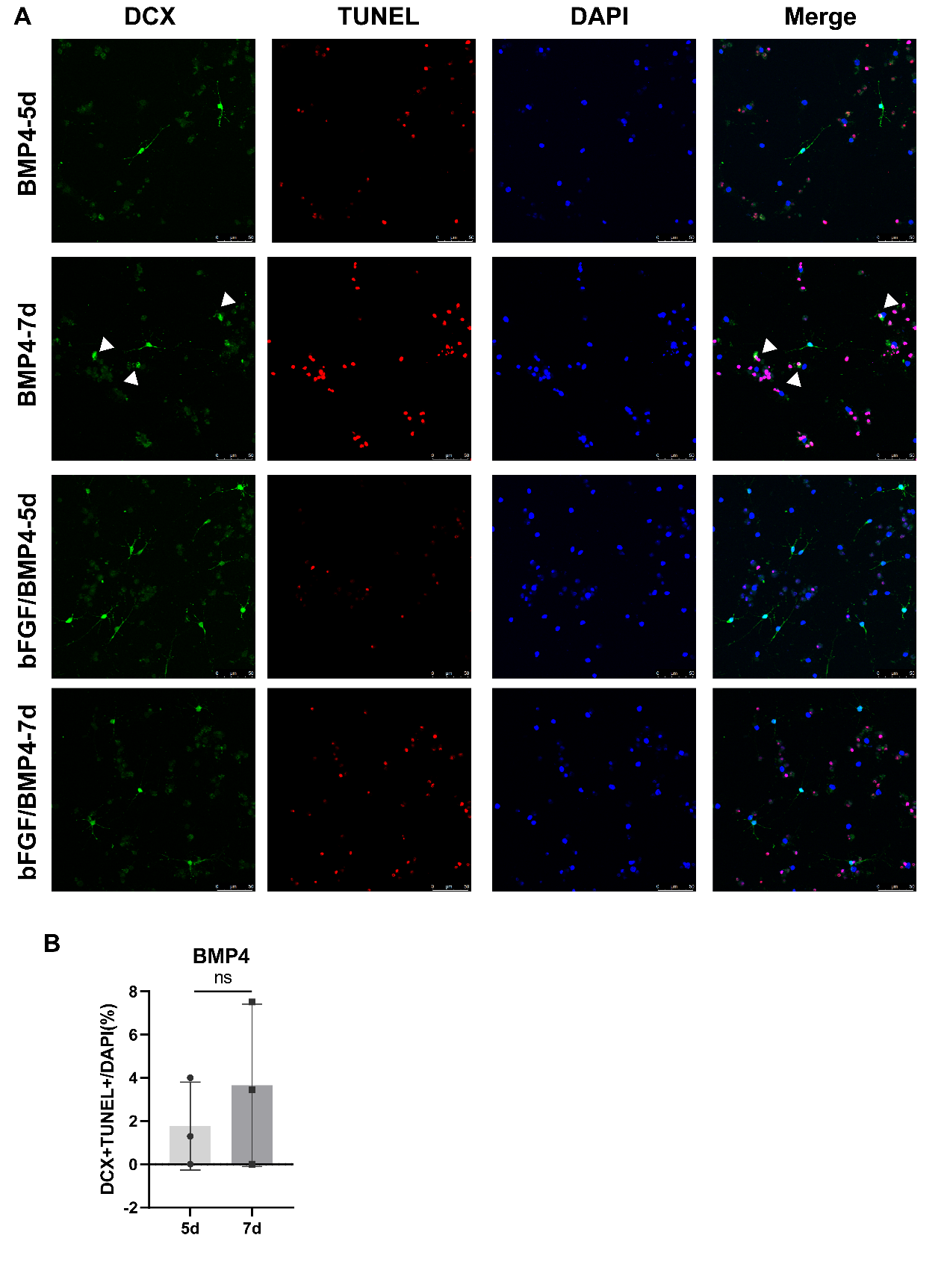


**Supplementary Figure S4.** The immunofluorescent staining of DCX^+^ an TUNEL^+^ on the 5th and 7th day of differentiation of BMP4- and bFGF/BMP4-treated NSCs. (**A**) The immunofluorescent staining of DCX^+^ an TUNEL^+^. The arrow indicates the
DCX^+^TUNEL^+^DAPI^+^ cells, and the cells with DCX in the cytoplasm were calculated. (**B**) Percentages of DCX^+^TUNEL^+^DAPI^+^ cells in (**A**), n=3, scale bar: 50μm, ns *p*>0.05.


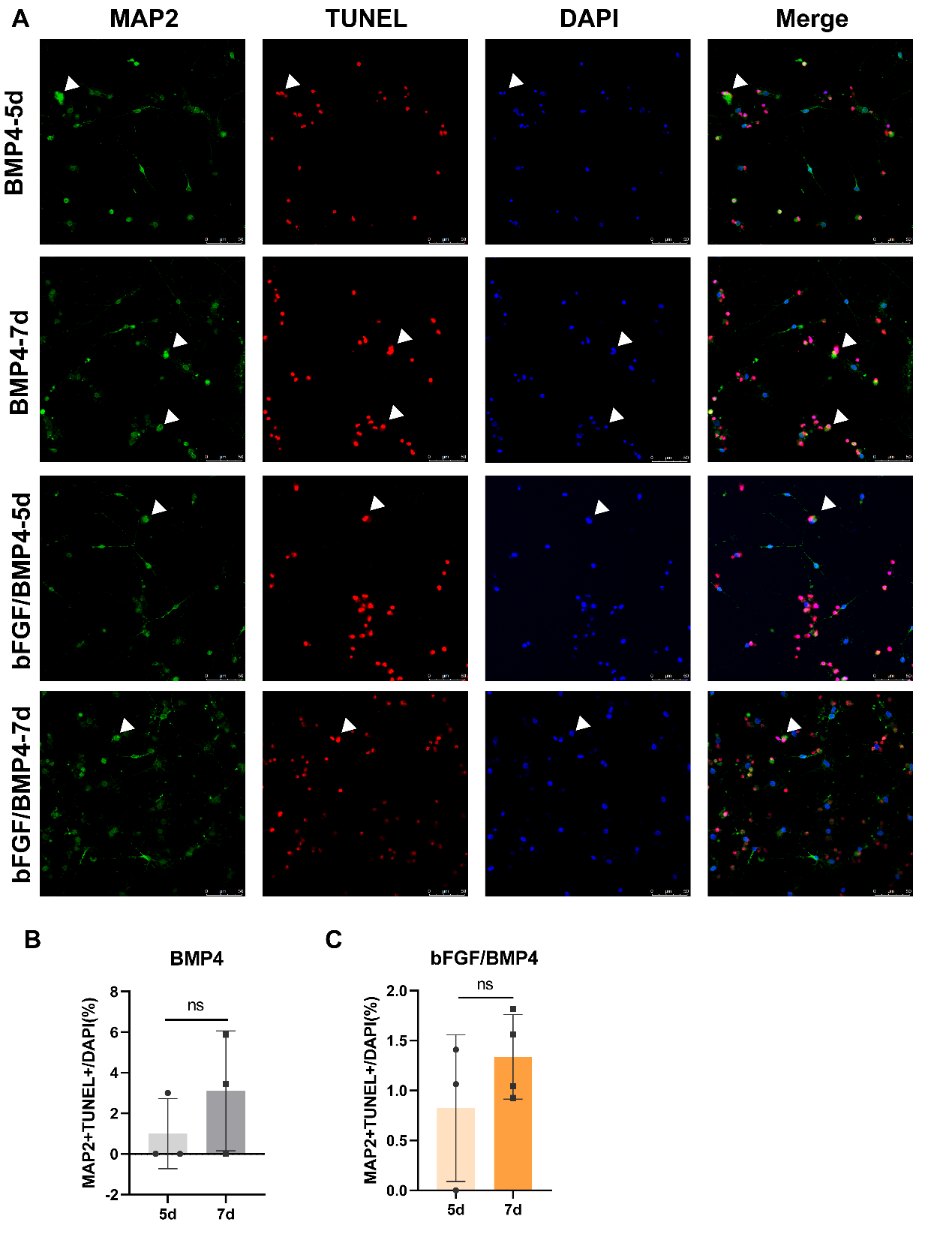


**Supplementary Figure S5.** The immunofluorescent staining of MAP2^+^ an TUNEL^+^ on the 5th and 7th day of differentiation of BMP4- and bFGF/BMP4-treated NSCs. (**A**) The immunofluorescent staining of MAP2^+^ an TUNEL^+^. The arrow indicates the
MAP2^+^TUNEL^+^DAPI^+^ cells. And the cells with MAP2 in the cytoplasm were
calculated. (**B**) Percentages of MAP2^+^TUNEL^+^DAPI^+^ cells in (**A**), n=3-4, ns *p*>0.05.

## Supplementary Tables

**Supplementary Table S1.** The marker gene set used in ssGSEA analysis.

**Supplementary Table S2.** Gene expression matrix of aNSC, qNSC2, qNSC2 and astrocytes used in the CIBERSORT analysis.

**Supplementary Table S3.** List of genes associated with cell cycle and chromatin segregation.

**Supplementary Table S4.** Markers of motile cilia and primary cilia.

**Supplementary Table S5.** The RNA row counts in our study. bFGF/EGF represent bFGF/EGF-treated NSCs, bFGF/BMP4 represent bFGF/BMP4-treated NSCs, and BMP4 represent BMP4-treated NSCs.
